# Sample Preparation and Separation of Lignans by Liquid Chromatography

**DOI:** 10.1002/jssc.70432

**Published:** 2026-05-05

**Authors:** Miikka Paloluoto, Susanne K. Wiedmer

**Affiliations:** ^1^ Department of Chemistry University of Helsinki Helsinki Finland

**Keywords:** anti‐inflammatory, antimicrobial, antioxidant, cytotoxicity, lignans, liquid chromatography, pinoresinol, sample preparation

## Abstract

Lignans are important phytochemicals found mostly in the resins of coniferous trees, but are also introduced into human diets through foods such as sesame seeds and extra‐virgin olive oil. They are pharmacologically interesting mainly because of their antioxidant activities, and they have also been shown to have cytotoxic and antimicrobial effects. Lignans are typically analyzed using liquid chromatography, although the complexity of the common matrices from which lignans are studied requires extensive sample preparation methodologies beforehand. While the simplest samples, such as wines and olive oils, can be analyzed without complicated procedures, most samples will require purification through liquid‒liquid extraction or silica gel column chromatography. Lignans are most often detected using mass spectrometry or ultraviolet‒visible spectrophotometry, but other methods, such as fluorescence and coulometric electrode array detectors, have also been proven useful. In this review, we will describe the typical characteristics of lignans with focus on their main pharmaceutical benefits, as well as typical sample preparation methodologies and liquid chromatographic analyses.

## 1 Introduction

Lignans are naturally occurring polyphenols, alongside compounds such as stilbenes, flavonoids, and phenolic acids, formed of two phenylpropanoid units. In nature, they are most commonly found in the resins, bark, and knots of coniferous trees, but can also be found in the branches, leaves, seeds, and fruits of several different tree species [1, 2]. In human diets, lignans are introduced through certain oilseeds, such as sesame and flax, olive oil, fiber‐rich foods such as cereals, and beverages such as tea, wine, and coffee [3]. Lignans are also a major component in certain plants that have been used in traditional medicines [4].

Lignan‐rich diets are known to have multiple health benefits, such as reducing the risk of cardiovascular disease and breast cancer [5, 6]. Due to their estrogen‐like structure, lignans are classified as phytoestrogens. Lignans are bioactive compounds that have been reported to have antioxidant, anti‐inflammatory, antiviral, and antifungal properties [7]. Several products are currently marketed, which contain lignan‐rich spruce resin as their active component, mostly different balms and ointments to treat small wounds on humans or animals [8]. There are also pills available for dietary lignan supplementation, promising the aforementioned cardiovascular and cancer‐inhibiting effects.

For the analysis of lignans from different sample matrices, high‐performance liquid chromatography (HPLC) is the most suitable technique. As lignans encompass polar and semipolar molecules, they can be separated well under common HPLC conditions. HPLC also requires no derivatization procedures before analysis, so less complex samples can be analyzed with minimal sample preparation. For the most complex samples, preparative or semi‐preparative HPLC can also be used as the last step in a sample preparation protocol before analysis using a suitable separation technique. This review provides an overview of sample preparation techniques and analysis of lignans, specifically focusing on their analysis using HPLC techniques. The paper includes a general discussion about lignans and their pharmaceutical effects, followed by sample preparation methodologies, including extraction and purification methodologies, and finally a discussion about HPLC stationary phases, eluents, and detectors suitable for lignan analyses.

## 2 Lignans

**FIGURE 1:**
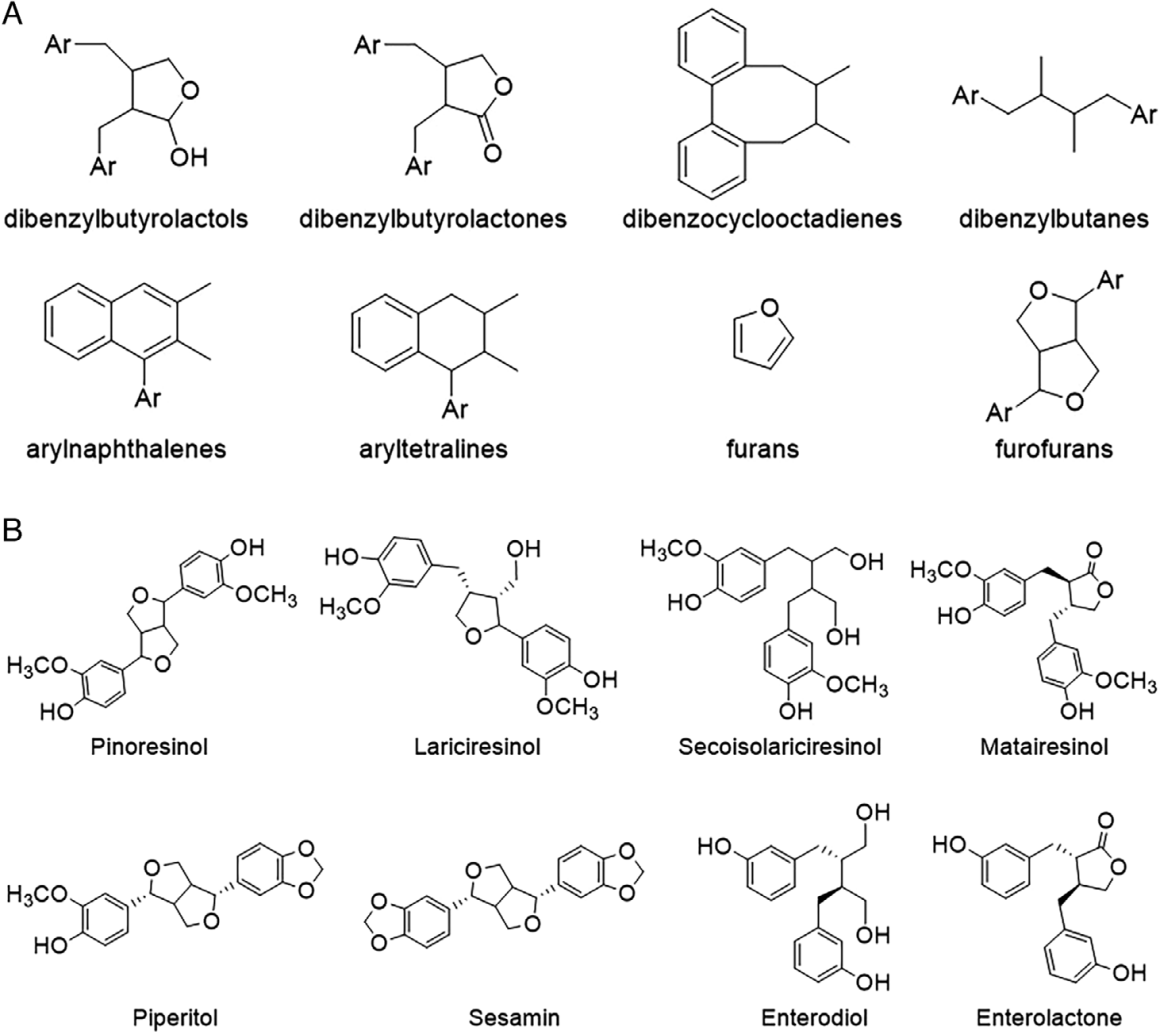
(A) Molecular structures of the backbones of the different lignan subgroups. Ar, aryl group. Structures without fixed aryl groups can have various substitutions on multiple different carbons. (B) Structures of the most common compounds appearing in text.

Lignans are dimers of phenylpropanoids coupled by the central carbons of the propyl sidechain (8,8′ position). If the monomers are linked via other carbons instead, they are considered neolignans. If one or more carbons are missing from the structure, the molecules are called norlignans. Condensation products, consisting of trimers (sesquilignans), tetramers (dilignans) or higher, are known as oligolignans. As the biosynthesis of lignans is directed by dirigent proteins and enzymes, certain isomers are often favored in nature over others, and common lignans found in enantiomeric excess are (+)‐pinoresinol, (+)‐lariciresinol, and (−)‐matairesinol (see Figure 1b). In plants, lignans typically appear either as lignan glucosides or as free aglycones.

Lignans can be categorized into subgroups based on the central structures linking them together. As shown in Figure 1, these subgroups are dibenzylbutyrolactols, dibenzocyclooctadienes, dibenzylbutyrolactones, dibenzylbutanes, arylnaphthalenes, aryltetralines, furans, and furofurans [5, 9]. Furofuran lignans, which are the most common subclass of lignans in lignan‐rich foods and plants, are characterized by a 2,6‐diaryl‐3,7‐dioxabicyclo[3.3.0]octane central skeleton structure [9]. The most common examples of naturally occurring furofuran lignans include pinoresinol, piperitol, and sesamin (see Figure 1b), although 137 different furofuran lignan molecules have been isolated from plants [9]. The most common sources of lignans in nature are bark [10, 11], knots [12, 13, 14, 15], and resins [16, 17, 18] of coniferous trees, as well as lignan‐rich grains and vegetables, such as flax and sesame seeds [19, 20], brassica vegetables, and extra‐virgin olive oil. They have also been found in decent amounts in foods not commonly associated with high lignan contents, such as multi‐grain bread, strawberries, and tea [19].

Lignans are biologically important phytochemicals and are integrated either as a structural motif in lignin polymers or as monomeric phytochemical constituents in plants, seeds, fruits, and plant exudates, such as palms and resins. Lignans exert a broad spectrum of biological activities in microorganisms, insects, animals, and humans, including growth inhibition, anti‐feeding, antimicrobial, antioxidant, and antifungal activities. Quantitation of lignans and their metabolites in various complex sample matrices is frequently required in studies focusing on the investigation of their interactions with various biological targets, the characterization of natural and synthetic lignin materials, the controlled degradation of lignins to biomass‐derived fine chemicals, and food supplement quality control of various dietary raw materials and derived products [14, 21]. Currently, the analytical quantification of minute concentrations of lignans in complex biological matrices is preferentially carried out by using HPLC in combination with mass spectrometric detection (LC‒MS), providing the benefits of high separation selectivity and sensitive and compound‐specific detection. However, in the presence of co‐eluting residual matrix components, MS detection often suffers from severe matrix effects, that is, signal enhancement or (more frequently) suppression relative to an uncompromised calibrant, making unbiased analyte quantitation challenging. While matrix effects in LC‒MS applications can, to some extent, be alleviated by optimizing sample preparation and chromatographic separation selectivity, these measures are elaborate, time‐consuming, and cost‐intensive. A more convenient strategy to address matrix effects consists of employing internal standardization with stable isotopically labeled target compounds. These stable isotopically labeled standards (SILSs) typically show chromatographic elution characteristics more or less identical to those of the target compound(s) and thus co‐elute with the latter but produce distinct MS signals due to isotopic substitution. However, with the analyte and the corresponding SILSs co‐eluting under given chromatographic conditions, both compounds experience possible ion enhancement/suppression effects simultaneously and to the same extent, both on the molecular ion and the fragment ion levels. Consequently, while matrix effects will affect the MS signals of the individual compounds, the relative analyte/SILS signal strengths of molecular ions and fragment ions remain unchanged and thus deliver “background‐corrected” mass abundance data for the unambiguous quantitation of the respective analyte concentrations. Another simple method for assessing matrix effects in MS detection is measuring matrix‐matched calibration curves by adding and measuring standard materials in the same matrix from which the target analytes are measured [22, 23].

The availability of high‐quality lignan standards is scarce, and the associated material costs are unaffordable for most academic research groups. For example, the pinoresinol standard materials available from commercial sources are typically sold for > €250/10 mg [24]. Moreover, despite the considerable costs, these materials are often only available at purity levels of 95%, which is not optimal for analytical standard applications. This is a topic that would benefit from additional research. The role of lignans in the life cycle of plants is still largely unclear. Most of this speculation is based on the biochemical properties, such as the antioxidant, antifungal, and antimicrobial activities, of lignans. For example, pinoresinol forms in plants when they are wounded, and it has been shown to provide antifeedant effects when ingested by caterpillars, protecting larvae from insectivorous ants [25]. When ingested by mammals, plant lignans are converted by the intestinal microbiota to the so‐called mammalian lignans enterodiol and enterolactone [26]. Initially, only secoisolariciresinol (see Figure 1b) and matairesinol were confirmed to convert to mammalian lignans, but pinoresinol, lariciresinol, sesamin, and hydroxymatairesinol are also known precursors [27] through studies on the effects of gut microflora on plant lignan using fecal incubation methods [28, 29].

### 2.1 Pharmaceutical Effects of Lignans

While lignans are generally known for their antioxidant capacities [11, 30, 31, 32, 33, 34, 35], they have shown poor results in 2,2‐diphenyl‐1‐picrylhydrazyl (DPPH) radical scavenging assays. Compounds such as flavonoids, tannins, and phenolic acids have shown better results in DPPH [31]. Li et al. measured seven lignans, as well as other phenolics, extracted from maple syrup, including lyoniresinol and secoisolariciresinol [31]. Coumarins and phenolic acid derivatives showed higher antioxidative effects than the measured lignans. Huang et al. applied ABTS (2,2′‐azino‐bis(3‐ethylbenzothiazoline‐6‐sulfonic acid) and ferric reducing antioxidant power (FRAP) assays alongside DPPH, in which both of the former had lignans as the strongest antioxidants of the measured analytes [33]. Lignans extracted from legume and sweet chestnut flours also performed well when measured with a FRAP antioxidant assay [32]. Lignans such as pinoresinol and matairesinol (see Figure 1b), which typically exhibit low performance in DPPH assays, demonstrate significant antioxidant activity in alternative tests such as liposome and low‐density lipoprotein (LDL) oxidation assays [11, 30].

The anti‐inflammatory effects of compounds are most often measured using in vitro tests to evaluate their effectiveness in inhibiting the production of proinflammatory compounds, most commonly nitric oxide [36, 37, 38]. However, tests can also be performed for prostaglandin E_2_ (PGE_2_) and tumor necrosis factor alpha (TNF‐α) [10, 11]. Pine bark lignans, specifically matairesinol and pinoresinol, have been shown to have anti‐inflammatory effects, albeit with varying results for the concentration values [11]. An experiment on human dietary intake of matairesinol has shown that this lignan reduces the probability of cardiovascular disease [39]. Of all the common plant lignans, pinoresinol has been shown to have the strongest effect in tests on human intestinal cells [40]. Secoisolariciresinol diglucoside (SDG) has often been the target in the search for anti‐inflammatory compounds [41, 42]. While SDG itself appears to have only weak effects, its metabolites secoisolariciresinol, enterolactone, and enterodiol show much stronger anti‐inflammatory properties [42].

The cytotoxic properties of various bioactive products, including lignans, have been well reported [43, 44, 45]. The effect of pinoresinol on breast cancer cells has been measured, and it has been demonstrated that it is more cytotoxic for cancer cells than for healthy cells, independent of the phytoestrogenic capacity of pinoresinol [46]. SDG and its metabolites, especially the mammalian lignan enterolactone, have also been studied for their effects on breast cancer cells [41, 47]. These studies, in which mice are typically fed a lignan‐rich diet after being injected with mammary cancer cells, often show enterolactone to be a potent cytotoxic agent against these cancer cells.

Many recent review papers can be found on the antimicrobial properties of phenols and lignans, see, for example, [48, 49, 50]. Barbary et al. found lignan‐rich flaxseed extracts to have significant effects on multiple different microorganisms, moderate effects on certain fungi, and anti‐hepatitis C activity at higher concentrations [51]. Certain rarer butane‐type lignans were shown to have effects on bacteria and fungi, whereas secoisolariciresinol did not have any effect on the investigated microbes [52]. Hakala et al. tested the extract of *Schisandra chinensis* against Chlamydia‐inducing microbes. The extracts were found to be nontoxic to human cells, and the lignan schisandrin B was identified as a growth inhibitor for microbes [53]. Flax root lignans were investigated as possible inhibitors for SARS‐CoV‐2, with the arylnaphthalene‐type lignans diphyllin and justicidin B being identified as the most potent inhibitors [54].

## 3 Sample Preparation

Sample preparation is one of the most crucial steps in the quantitative analysis of molecules from complex matrices. While certain liquid samples can be analyzed with minimal preparation, most samples require preparation beforehand, no matter which separation technique is chosen. As sample preparation typically results in improved chromatographic resolution and selectivity, a cleaner sample can be analyzed with more efficient methods, allowing for faster and more solvent‐efficient analysis. The importance of sample preparation cannot be overlooked, as several issues can occur during analysis if the sample preparation is insufficient. If nontarget compounds co‐elute at the same time as the target analytes, they can distort analyte peaks, making them useless for analysis without deconvolution methods such as mass spectrometry (MS). Sample preparation also reduces matrix effects, which occur when matrix compounds inhibit the detection of target compounds. In the worst case, matrix components can become stuck in the column, resulting in increased backpressure and leading to changed chromatographic profiles (retention time, peak area) with every injection. A wide range of sample preparation methodologies for lignans is available, see, for example, [14, 34, 55]. Below, these are briefly discussed based on the type of matrix. Notably, only such sample preparation methodologies that have been used for the extraction or purification of lignan samples are considered here.

### 3.1 Solid Sample Extractions

The first step after acquiring a solid sample, before any extraction, is the drying and storage of the sample. For most plant materials, the sample is separated into different parts, for example, separating the inedible parts of fruits from the edible ones, after which the sample is dried before it is homogenized. The drying of samples is sometimes done by simply air‐drying, but most often, lyophilization and freeze‐drying techniques are utilized. Samples are most often stored either in refrigerators or freezers to avoid the loss of volatile sample components. Before extraction, the sample is homogenized by grinding into a powder to increase the efficiency of the extraction. When assessing bulk phenolic compounds, it might be essential to store the samples in the dark, as some of these compounds are known to photoisomerize quite easily [56, 57]. However, such stability issues are typically not discussed in the literature.

Examples of extractions of solid plant samples are provided in Table 1 [12, 13, 15, 33, 58, 59, 60, 61, 62, 63, 64, 65, 66, 67, 68, 69, 70, 71, 72, 73, 74, 75, 76, 77, 78]. The most common method for extraction is maceration, where the sample is simply soaked in an organic solvent for an extended period. In addition, reflux techniques, such as Soxhlet and continuous hot extraction, and ultrasonication‐assisted extraction (UAE), are common. Almost all the sample extractions listed in Table 1 are followed by further processing with liquid‒liquid extraction (LLE) or solid‐phase extraction (SPE).

**TABLE 1:** Selected examples of lignan extractions from solid samples using several different methods.

Species	Part	Method	Solvent	Time	Temp	Sample–solvent ratio	References
Several foods	several	Incubation	70% MeOH, 0.3 M NaOH	1 h	60°C	1:24	[73]
*Pinus halepensis*/*Pinus sylvestris* (tree)	multiple	Maceration	70% MeOH	3 h	RT	1:5	[59]
*Punica granatum* L. (pomegranate)	Fruit	Maceration	Acetone:0.01 M HCl 80:20	1 h	60°C	1:30	[65]
*Helicrysum melaleucum* (plant)	Multiple	Maceration	MeOH	24 h	RT	1:10	[67]
*Triticum aestivum* L. (wheat)	Seed	Maceration	80% EtOH	3 h	60°C	1:8	[64]
*Linum usitatissimum* L (flax)	Seed	Maceration	40% EtOH	24 h	RT	1:2	[66]
*L. usitatissimum* L. (flax)	Seed	Maceration	80% MeOH	4 h	55°C	1:20	[74]
Several foods	Several	Maceration	70% MeOH, 0.3 M NaOH	1 h	60°C	1:24	[70]
*Forsythia intermedia* (shrub)	Stem	Maceration	MeOH	1 h	50°C	1:20	[68]
*Bombax ceiba* (tree)	Root	Percolation	95% EtOH	—	RT	1:10	[77]
*Diospyros kaki* Thunb. (persimmon tree)	Fruit/leaves	Sequential percolation	95% EtOH, 50% EtOH	4 days	RT	1:10	[33]
*Cinnamomum camphora* (tree)	Leaves	Reflux	95% EtOH	2 h	BP	1:2	[78]
*Polyscias guilfoylei* (shrub)	Stem	Reflux	EtOH	6 h	BP	1:4	[58]
*Melia azedarach* L. (tree)	Fruit/leaves	Sequential Soxhlet	Hexane, EtOH	—	BP	—	[60]
*Pinus cembra* (tree)	Knots	Sequential Soxhlet	Hexane, EtOH, 95% acetone	6 h	BP	1:50	[12]
Coniferous trees (4 species)	Knots	Sequential Soxhlet	Hexane, Acetone	8 h	BP	—	[13]
Coniferous trees (4 species)	Knots	Sequential Soxhlet	Hexane, 95% Acetone	8 h	BP	—	[15]
*Tilia cordata* (tree)	Fruit	Sonication	70% MeOH	1 h	—	1:40	[61]
*Forsythia suspensa* (shrub)	Leaves	Sonication	20% DES (also 70% MeOH/EtOH)	30 min	30°C	1:30	[69]
Grape	Seed	Sonication	90% MeOH	5 min	RT	1:50	[63]
*Abies alba* Mill/*P. sylvestris* (tree)	Wood	Sonication	MeOH	2 h	60°C	—	[72]
Several foods	Several	Sequential ASE	Hexane, Acetone, 70% Acetone	5 min	90°C	—	[75]
Coniferous trees (4 species)	Knots	PLE	Hexane, 95% Acetone	15 min	100°C	1:4	[15]
Tunisian olives	Leaves	MAE	MeOH or EtOH 40%–100%	6 min	80°C	1:8	[76]
*Pinus pinaster* (tree)	Knots/stems	SFE	CO_2_ with 10% EtOH	—	50°C	—	[62]
Coniferous trees (4 species)	Needles	HTE	Water or EtOH	3 h	120°C	1:10	[71]

Abbreviations: ASE, accelerated solvent extraction; DES, deep eutectic solvent; EtOH, ethanol; HTE, hydrothermal extraction; MAE, microwave‐assisted extraction; MeOH, methanol; NaOH, sodium hydroxide; PLE, pressurized liquid extraction; RT, room temperature; SFE, supercritical fluid extraction.

The choice of solvent plays a crucial role in the recovery and selectivity of any given extraction technique. In lignan analysis, especially considering food samples, an initial defatting extraction step with hexane is often performed. This removes from the sample high molar mass components, which are often not of interest and could cause matrix effects or damage to the analytical column. The solvent of choice for extracting lignans from complex matrices is methanol (MeOH), often as a mixture with water at a percentage of 70%–80%. As lignans and other phenolic compounds are decently polar molecules, MeOH provides the best recovery, although it is also the least selective solvent. Less polar solvents, such as ethanol and acetone, are also sometimes used [10, 79].

Maceration is the most commonly used extraction technique in lignan analysis due to the simplicity of the procedure [64, 66, 68]. It involves simply immersing the powdered sample in an organic solvent and stirring. Maceration is mostly performed at room temperature, but sometimes temperatures near the boiling point are used to speed up the extraction. The main downsides of this technique are the time and solvent requirements, as well as the lack of selectivity. The typical requirements for one of these extractions are, at a minimum, 1 h per extraction at an elevated temperature, with sample–solvent ratios typically approximately 1 to 10. When MeOH is used as the solvent, it will extract nearly all low and medium‐molar mass compounds from the matrix, giving an extract that will inevitably require extensive processing afterward.

Maceration has been applied to extract lignans from tree samples from different parts of various species. They are typically extracted using more polar solvents, such as MeOH, acetone, or ethanol (EtOH), which tend to be less selective and extract most of the compounds from the sample. The extractions are mostly performed at room temperature, which is often time‐consuming; sometimes, a single extraction can take a whole week [64]. Food samples are also often extracted using maceration, as the compounds in food samples are typically easy enough to extract without more complex procedures. Examples of foods that have been extracted in this way include artichoke, pomegranate, seeds from wheat and flax, and cinnamon. The solvents of choice remain the alcohols MeOH and EtOH, mostly as aqueous mixtures. It is more common to apply heating to food samples, in which case the time required for extraction can be reduced to 1–4 h, with extractions at room temperature still taking up to a day to perform [19, 65].

Percolation is another conventional extraction technique that is less commonly used but has applications in large‐scale or industrial extractions. The solvent is poured on the top of the sample and allowed to flow through the ground sample matrix. These extractions can take multiple days to complete, and as the time depends on the solvent flowing through the sample, the process cannot be sped up by raising the temperature above room temperature. However, it is still the most useful method for extracting samples in multi‐kilogram amounts.

Percolation was used to extract unknown lignans from the roots of *Bombax ceiba*, with 100 L of 95% EtOH applied to 10 kg of the dried and powdered substrate at room temperature. After extraction, 300 g of residue remained for purification and isolation of compounds [77]. An even larger scale extraction was performed to extract lignans from persimmon leaves, with 50 kg of dried and milled leaves first being extracted with 600 L of 95% EtOH, then an additional 500 L of 50% EtOH. The process yielded 4.5 kg of residue, which was then heavily processed to isolate the compounds [33].

Reflux extraction techniques, such as Soxhlet or continuous hot extraction, are commonly used for smaller sample amounts. In these methodologies, the solvent is boiled from the receiving flask to a condenser above the sample, bringing fresh solvent constantly into the sample vessel. This method saves both the solvent, which is recycled endlessly, and time, as the condensed solvent will be near its boiling point when in contact with the sample. It is common in reflux extractions to separate analytes categorically by performing sequential extractions with a low polarity solvent and then using a solvent with higher polarity to make it easier to purify the target analyte for the target fraction.

The most common procedure for lignans involves first using hexane to clean the sample from the lipophilic matrix components, which would interfere with analysis. Afterward, the target analytes are extracted with a more polar solvent such as EtOH or acetone, typically in a single step, but sometimes up three‐step extractions are also performed. A stronger solvent, such as MeOH, is most likely not commonly employed, as the harsher condition of exposing the sample to a solvent at its boiling point for multiple hours provides good enough performance with weaker solvents. Soxhlet methods have been used to extract antifungal agents from chinaberry tree samples, first defatting with hexane and then extracting the target compounds with EtOH [60]. Soxhlet extraction using EtOH without prior defatting in a study on antioxidative compounds in *Polyscias guilfoylei* required sequential LLE for the extract containing high‐molar‐mass molecules [58].

Ravetti Duran et al. conducted a study comparing different extraction methods to recover the valuable compounds remaining in industrial wood waste [14]. They compared maceration, Soxhlet, simulated countercurrent, and percolation, with the goal of determining which method would be applicable for industrial‐scale operations. While percolation offered the lowest yield out of all the techniques, it was singled out as an attractive option due to both the ease of integration and the scalability of the method.

UAE is a technique similar to maceration in that the sample is directly immersed in the solvent, but the extraction is accelerated by ultrasonication. The ultrasonic waves form rapidly expanding and compressing bubbles in the solvent, which eventually reach a critical point and explode in cavitation with extreme temperatures and pressures, effectively breaking down the sample matrix for more efficient extraction. UAE is most often performed at room temperature, as sound waves propagate better at lower temperatures. A study by Cittan et al. comparing the efficiency of UAE against traditional infusion extraction (essentially maceration with near‐boiling ultrapure water) for the extraction of phenolic components from *Tilia cordata* fruits showed that both techniques had similar efficiencies [61]. While both extractions were allowed to run for 1 h, the UAE method with 70% MeOH provided 50% greater yields and managed to extract certain phenolic components that remained in the matrix during the water extraction. Credit was given to the infusion process for its simplicity, while still managing to extract significant amounts of phenolic compounds.

Lignan reference materials were isolated from the branch wood of *Abies alba* Mill. and *Pinus sylvestris* L. by Patyra et al., by first defatting the samples with hexane and then using UAE with pure MeOH as the solvent [72]. The extractions were performed at 60°C, three times for 2 h each, and yielded reference materials for 11 different lignans after extensive purification using silica column chromatography (CC) and preparative liquid chromatography (prep‐LC). In a recent study, Liu et al. developed a method to extract lignans from the leaves of *Forsythia suspensa* using deep eutectic solvents (DES) in ultrasonication extractions [69]. DESs consist of a mixture of a hydrogen bond acceptor and a donor, which will form special hydrogen bond structures with the analytes, facilitating extraction. The main downside noted for DES is the difficulty in separating the target analytes from the DES after the extraction. In their work, a DES of choline chloride‐ethylene glycol (1:3) mixed with 20% water proved optimal for UAE, from which the extract was processed with a macroporous HPD100 resin in 70% EtOH to separate the lignans from the DES solution.

**FIGURE 2:**
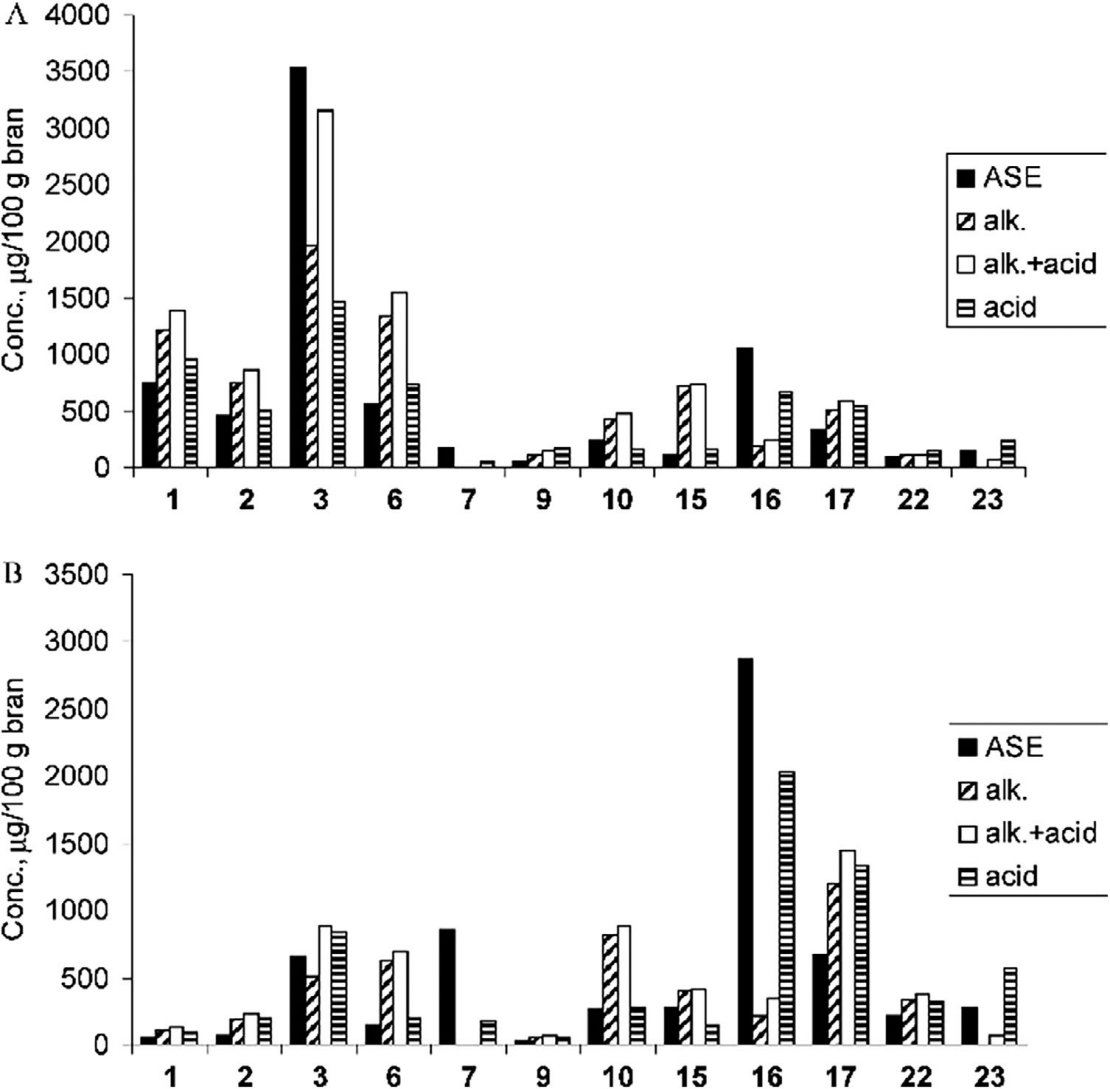
Concentrations of lignans extracted from (A) rye bran and (B) wheat bran using ASE in alkaline (alk.), acidic (acid), and alkaline followed by acidic (alk. + acid) conditions, measured using HPLC–MS/MS. (1) (+)‐Pinoresinol, (2) (+)‐medioresinol, (3) (+)‐syringaresinol, (6) (+)‐lariciresinol, (7) iso‐hydroxymatairesinol, (9) cyclolariciresinol, (10) (−)‐secoisolariciresinol, (15) (−)‐matairesinol, (16) (−)‐7‐hydroxymatairesinol, (17) 7‐oxomatairesinol, (22) todolactol A, (23) α‐conidendrin. Figure adapted from Peñalvo et al. [73] with permission from American Chemical Society.

In accelerated solvent extraction (ASE), the extraction is performed inside a specialized tube capable of withstanding high pressures. The solvent is brought to the boiling point, and pressure is applied using an inert gas, reducing both the amount of time required for the extraction and the volume of solvent required while also increasing the yields. ASE has been applied in the analysis of lignans from cereals, nuts, and oilseed while comparing its performance to simple acidic and alkaline extractions [75]. While a combination of acid/alkaline extraction provided better yields overall, ASE could extract 7‐hydroxymatairesinol, a compound that is unstable under both acidic and alkaline conditions, from the analyzed cereals. The extracted concentrations of several lignans using these techniques are shown in Figure 2.

When solvents are heated using heating mantles or water baths, the heating applied is not uniform, with the center of the solvent being cooler than the solvent closer to the walls of the vessel. This can be remedied by using microwave‐assisted extraction (MAE), where the heating comes from microwaves and is thus applied uniformly throughout the solvent. An optimized procedure was developed to extract lignans from olive leaves using MAE, and this extraction gave better yields in 6 min compared to the 24 h conventional extractions performed [76].

One common problem with most extraction methods is that the solvents used are typically harmful, and they are sometimes used in excessive quantities. A method that aims to solve this problem is supercritical fluid extraction (SFE), where supercritical CO_2_, which is considered more environmentally friendly than most common organic solvents, is used as the solvent. A SFE method was developed to extract lignans from *Pinus pinaster* wood and then optimized by adding ethanol as a cosolvent [62]. Another possibility includes the use of subcritical water extraction, as performed in a recent study on the extraction of polyphenols and lignans from silver fir (*A. alba*) bark extracts [34].

Another option for environmentally friendly extractions is to replace harmful organic solvents with water. Hydrothermal extraction (HTE), also sometimes known as pressurized hot water extraction, is a technique similar to ASE developed for this purpose. Similar to ASE, HTE centers around applying both heating and pressure to the extraction vessel to accelerate the process. HTE has been applied to extract lignans, as well as other extractables, from the needles of coniferous trees [71]. It was found that water worked well as a solvent for phenolics such as lignans, while switching the solvent to ethanol increased the yield of high‐molar‐mass components such as lipids and polyphenols.

Table 1 [12, 13, 15, 33, 58, 59, 60, 61, 62, 63, 64, 65, 66, 67, 68, 69, 70, 71, 72, 73, 74, 75, 76, 77, 78] shows the procedures used to extract lignans from various matrices using different techniques. A general trend is that the more conventional the method used is, the longer the extraction takes. With more advanced instruments utilizing high temperatures and pressures, the extraction times can be decreased dramatically. Of note is also the sample–solvent ratios for sonication extractions, as while the technique provides rapid extractions, only small amounts of the solid matrix can be processed in a single run.

### 3.2 Purification of Extracted Samples

An extracted sample will contain a large amount of matrix components, even after sequential extractions, which fractionate the sample at the extraction step. If only a certain class of compounds is being investigated, they should be separated from the rest before analysis. Especially in quantitative analysis, purification procedures are often employed after extraction. Selectivity is key, as the method employed should result in a sample with as high a recovery for the target analytes as possible, with minimal amounts of matrix components. Table 2 [12, 33, 58, 64, 65, 67, 75, 80, 81, 82] shows examples of purification procedures that followed lignan extractions from different solid samples. As lignans are often found in nature chemically bonded in larger structures, typically as diglucosides or in lignin polymers, it is sometimes appropriate to free the lignans from these structures using hydrolysis techniques. The most common method involves using glucuronidase and sulfatase enzymes purified from the snail digestive juice *Helix pomatia* under slightly acidic sodium acetate buffer conditions. Hydrolysis can also be performed simply using acid or base, although harsher conditions have the possibility of producing compounds that were originally not found in the matrix, such as anhydrosecoisolariciresinol formed from secoisolariciresinol during acid hydrolysis [64].

**TABLE 2:** Extraction of lignans from solid samples with the purification procedures that followed.

Sample	Extraction	Purification	Analysis	References
Cereals, oilseeds, nuts	ASE: hexane → acetone → 70% acetone	Enzymatic hydrolysis → LLE with EA	LC‒MS/MS	[75]
Cinnamon	Maceration: 95% ethanol	LLE: EA → silica CC and SPE → prep‐LC	UV, NMR, MS, IR	[82]
*Helichrysum melaleucum*	Maceration: methanol	Filter and inject	LC–UV/MS	[67]
Knotwood (Swiss pine)	Soxhlet: hexane, ethanol, 95% acetone	Filter and inject	LC‒MS	[12]
Persimmon leaves	Maceration: 95% ethanol, 50% ethanol	LLE: PE, EA → silica CC and SPE → prep‐LC	LC‒MS/MS	[33]
*Polyscias guilfoylei*	Reflux: ethanol	LLE: hexane, dichloromethane, EA	NMR, MS, IR	[58]
Pomegranate	Soxhlet: 80/20 acetone/0.01 M HCl	Acid hydrolysis → LLE: 1/1 hexane/EA	LC–DAD/MS	[65]
Sesame seed	ASE: hexane → acetone → 70% acetone	Enzymatic hydrolysis → LLE with EA	LC–UV/MS	[80]
*Vibrunum erosum*	Maceration: 80% methanol	LLE: EA, butanol → silica CC and SPE	NMR, MS, IR	[81]
Wheat	Maceration: hexane → 70% EA	Acid hydrolysis → LLE: 1/1 hexane/EA	CE–MS	[64]

Abbreviations: CC, column chromatography; EA, ethyl acetate; LLE, liquid‒liquid extraction; SPE, solid‐phase extraction.

Dinelli et al. conducted a study on the differences in lignan contents between new and old cultivars of Italian soft wheat using CE–MS detection [64]. They found pinoresinol and secoisolariciresinol in all cultivars, but the lignans arctigenin, hinokinin, and syringaresinol were found only in the old cultivars. During the study, they analyzed both hydrolyzed and non‐hydrolyzed wheat samples, with the non‐hydrolyzed samples containing no aglycones and only derivatives of lignans detected.

In a paper concerning the lignan contents of pomegranates, Fischer et al. also performed optimizations on their hydrolysis procedures to select the more effective method between acid and enzymatic hydrolysis [65]. They found that alkaline hydrolysis followed by enzymatic cleavage in MeOH was unsuitable in this case, while an acidic enzymatic hydrolysis procedure in MeOH gave a worse yield compared to purely acidic hydrolysis. For their purposes, the optimal procedure involved extracting the pomegranate samples with 80% acetone acidified with 0.01 M HCl.

Once the hydrolysis is finished and the lignans are released from the high molar mass matrix structures or once the aglycones are obtained from the glucosides, the target compounds are typically extracted by LLE. The organic solvent in the extract is evaporated under vacuum, and the remaining sample is dissolved or diluted with water. The aqueous fraction is then extracted with organic solvents immiscible with water, for example, hexane, acetone, or ethyl acetate (EA), with the compounds migrating to the organic phase depending on their affinity with that solvent. As lignans are medium‐polar molecules, they are most often extracted into medium‐polar organic solvents. The complexity of the LLE procedure depends on the complexity of the extraction and the requirements of the analysis at hand. Characterization studies, where the presence of compounds is identified in any given matrix, will demand less purification of the sample. After sequential extractions, the analytes are already separated into categories based on their polarities, and a single LLE step is typically used after the hydrolysis step [75, 80].

If the sample is not defatted during extraction, it is usually done during the LLE step. This works similarly to sequential extraction, where the aqueous phase containing the sample components is first extracted with a lipophilic solvent such as hexane or petroleum ether (PE). A medium‐polar solvent such as EA or acetone is then used to extract the target lignans from the aqueous phase, as analytes in an organic solvent are easier to either process for further purification or evaporate and reconstitute in a solvent suitable for HPLC [33, 58].

The next step of purification after LLE will usually be chromatography, either on silica gel or using commercial SPE cartridges. For silica gel CC (silica CC), which is a polar stationary phase, the solvent gradients begin with lipophilic solvents such as hexane, which elute the highly lipophilic matrix components, while medium‐polar lignans are retained in the column. To elute the target compounds from the column, the proportion of more polar solvents such as EA or MeOH is gradually increased until all the analytes are released. The principle is the same in C18‐SPE, except that the stationary phase is coated with lipophilic carbon chains, and therefore, the polarities in the mobile phase gradient elution are reversed. Cartridges are also available for other retention mechanisms, such as ion exchange or size exclusion.

Pinoresinol and other phenolic compounds have been purified and isolated from medicinal herbs using combinations of silica CC and SPE methods. The research group of In et al. processed the EA LLE fraction of the MeOH extract of *Viburnum erosum* by first subjecting the extract to silica CC with a hexane/EA gradient and then a chloroform/MeOH/water gradient, yielding 21 fractions [81]. These fractions were then further purified with silica CC, reversed‐phase ODS CC, and size‐exclusion SPE using Sephadex LH‐20 medium as the stationary phase. The process yielded 10 purified compounds for characterization with NMR, MS, and IR. A similar procedure was utilized for an EtOH extract of *P. guilfoylei*, where a silica CC procedure using a dichloromethane/acetone gradient gave 13 fractions for further processing [58]. After an extensive purification process with size‐exclusion SPE and both normal and reversed‐phase CC, 11 compounds, including pinoresinol, were isolated.

If individual compounds need to be isolated from the sample matrix, prep‐LC is sometimes needed even after LLE and silica CC or SPE. Huang et al. isolated secoiridoids and lignans from the EtOH extract of cinnamon [33]. After LLE with PE and EA, purification with CC on both silica and polyamide columns, as well as SPE on Sephadex LH‐20, the final steps of the purification for each compound involved a reversed‐phase prep‐LC method, utilizing both UV and refractive index detection. A total of 21 compounds were isolated and quantified, including pinoresinol.

Lignans and neolignans from the bark of *Machilus robusta* have been extracted for in vitro assays against HIV‐1 replication. [83] An EtOH extract of ground bark was dissolved in water, extracted with EA, and then subjected to silica CC on a PE‐EA gradient. Fractions from the CC were then further purified with size‐exclusion SPE on Sephadex LH‐20. One compound was isolated during SPE, and the rest were obtained either by preparative high‐performance thin‐layer chromatography or reversed‐phase prep‐LC. A total of 16 compounds were isolated, which were then characterized with IR, NMR, and MS, as well as used for several different biological assays. Pinoresinol and lyoniresinol were found to be active against serum deprivation‐induced cell damage.

Sometimes purification is not needed, and an extracted sample can simply be filtered before injection. This is mostly done with more experimental methods, to see which analytes these methods manage to extract, but it can also be done with sequential extractions, when using a solvent weaker than MeOH, or with liquid samples where most of the compounds are already water‐soluble. Injections of unpurified samples, especially from more complex matrices, are unsuitable for quantitative analysis due to increased matrix effects, and there is also the possibility that repeated injections will cause damage to the analytical column.

The main use cases for “filter and inject” methods for solid samples are profiling studies, where the aim is to qualitatively identify as many compounds present in the sample as possible. Analyzing complex samples requires the use of a sensitive detector, such as MS, to minimize the overlap between the peaks. In a study profiling the different phenolic compounds, such as phenolic acids, flavonoids, and lignans, from the MeOH extracts of *Helichrysum melaleucum* samples from different locations, the extract was filtered, and 68 phenolic compounds were detected and characterized by HPLC–DAD/ESI‐MS*^n^* [67]. The contents of phenolic compounds in the samples seemed to depend on the altitude from which the samples were taken, especially pinoresinol and flavone derivatives, which could serve as geographical markers.

Coniglio et al. investigated the yellowing of water‐based coatings on knotwood [12]. The samples from both the *Pinus cembra* knots and the aged coating applied to the knots were injected into LC‒MS after filtering. Nine compounds, five of which were lignans, were detected both in the knotwood sample and the coating exposed to the knots. The authors suspected that the discoloration of the coating was caused by the formation of stilbene derivatives that form when the coatings come in contact with the knots.

### 3.3 Extraction of Liquid Samples

As the compounds in liquid samples are already dissolved in a solution, conventional extractions with organic solvents are unnecessary. With certain analyses, such as analysis of multiple compounds using HPLC, it is viable to either dilute aqueous samples or dissolve oils in a solvent suitable for analysis and then simply filter the liquid sample before injection. Although direct injection can be used, purification by either LLE or SPE is often required for the analysis of lignans in liquid samples. Table 3 [3, 22, 23, 27, 63, 84, 85, 86, 87, 88, 89, 90, 91] shows the different treatments given to various liquid samples in lignan analysis before injection into an analytical instrument.

**TABLE 3:** Purification of liquid samples for lignan analysis.

Sample	Purification	Analysis	References
Coffee	Enzymatic hydrolysis → filter and inject	LC‒MS/MS	[22]
Flaxseed oil	Diol‐bonded SPE	LC‒MS	[86]
Olive oil	Dissolve in hexane → LLE with 6/5 methanol/water	LC–UV	[84]
Olive oil	Dissolve in hexane → LLE: 60% methanol	LC–FLD	[23]
Olive oil	Dissolve in hexane → LLE: 60% methanol	LC–DAD/MS	[91]
Olive oil	Dissolve in hexane → diol‐bonded SPE	LC–DAD/MS	[87]
Plasma and urine	Enzymatic hydrolysis → C18‐SPE	LC‒MS/MS	[89]
Plasma	Enzymatic hydrolysis → LLE:DEE → ion‐exchange SPE	LC–CEAD	[90]
Serum	Enzymatic hydrolysis → C18‐SPE	LC‒MS/MS	[85]
Urine	Enzymatic hydrolysis → LLE:DEE → ion‐exchange SPE	LC–CEAD	[27]
Wine	Dilute with 0.01% acetic acid, filter and inject	LC‒MS	[63]
Wine	C18 SPE → enzymatic hydrolysis → LLE: DEE	LC–CEAD	[88]

Abbreviations: DEE, diethyl ether; LLE, liquid‒liquid extraction; SPE, solid‐phase extraction.

#### 3.3.1 Oils and Beverages

The extraction process of olive oil before LC analysis typically involves dissolving the olive oil in hexane, after which LLE is performed with a 50%–60% solution of aqueous MeOH [23, 84, 91]. The extract was then evaporated under nitrogen, reconstituted in MeOH, and filtered before injection. Another method uses SPE with a diol phase to retain the polar components of olive oil dissolved in hexane in the SPE stationary phase [87]. Before loading the oil, the cartridge is first conditioned with MeOH to wash the stationary phase and then with hexane to prepare it for loading. The nonpolar components are first eluted with hexane, then 90:10 hexane/EA, after which the polar compounds are collected by eluting the column with MeOH. The extract was then evaporated under nitrogen and reconstituted before analysis. A similar procedure using DSC‐Diol SPE has been utilized for the analysis of flaxseed oil [86].

For wine samples, the complexity of sample preparation depends mainly on the detector. An LC‒MS analysis comparing lignan amounts in three different types of wine was performed simply by diluting the samples with 0.01% acetic acid and then filtering and injecting the sample [63]. An LC analysis using a coulometric electrode array detector (CEAD), on the other hand, required more extensive preparation [88]. The samples were first purified with C18‐SPE, after which hydrolysis was performed both enzymatically and with acid. The samples were then extracted with diethyl ether, combined, and finally subjected to ion‐exchange chromatography using QAE‐Sephadex. While all the wines they analyzed had similar lignan profiles, the white wine samples had lower total quantities of lignans.

Sample preparation for brewed coffee and tea has been performed using methods of varying levels of complexity [3, 22]. For gas chromatography (GC) analysis, the procedure involved multiple hydrolysis, ether extraction, and SPE, as well as the mandatory derivatization needed when performing GC‒MS of polar molecules [3]. A paper by Angeloni et al. compared three simple preparations for espresso coffee using LC‒MS/MS as the detector: dilute and filter, acidic, and enzymatic hydrolysis [22]. After testing multiple conditions and different enzymes, enzymatic hydrolysis with the enzyme mixture Clara‐diastase, which is often used to digest food samples, provided the best recovery from the coffee sample.

#### 3.3.2 Biofluid Samples

**FIGURE 3:**
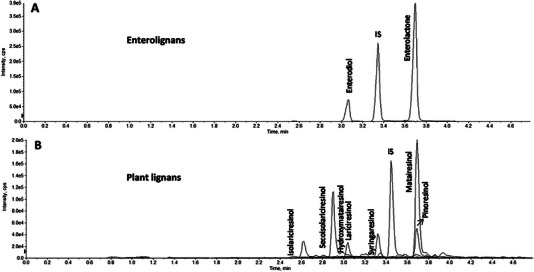
LC‒MS multiple‐reaction monitoring (MRM) chromatogram from the analysis of a human plasma sample, showing (A) enterolignans and (B) undigested plant lignans. Separation was achieved using an Ascentis C18 column, with gradient elution using mobile phases consisting of water + 0.1% formic acid and ACN + 0.1% formic acid. Figure adapted from Nørskov et al. [89] with permission from American Chemical Society.

The lignans of interest in biofluid analysis are the so‐called mammalian lignans, enterolactone and enterodiol, instead of the plant lignans found in most other samples, as research typically focuses on the digestive system's ability to convert plant lignans into mammalian lignans. Enzymatic hydrolysis using β‐glucuronidase, followed by C18‐SPE, is also popular for serum [85], plasma [89], and urine [89], as these are easy to perform on a larger scale using well plates to perform multiple extractions at once. Once the cartridges are loaded with the sample, they are washed with aqueous MeOH, after which the target compounds are released using nonpolar organic solvents such as acetonitrile (ACN) or EA/ACN. Figure 3 shows an MS run for a human plasma sample, which contains both the converted enterolignans and the remaining unconverted plant lignans in the plasma. [89] In this study, both humans and pigs were fed a lignan‐enriched diet, and LC‒MS was used to quantify the plasma lignan content in the range of 0.024–100 ng/mL. The lignan concentration in human hydrolyzed plasma was < 67 nm for enterolignans and < 1 nm for enterolignans. C18‐SPE was used for the cleanup of the samples. The authors noticed that the concentration of enterolignans in plasma (and urine) from both humans and pigs was higher compared to plant lignans, which agrees with the current understanding that the majority of plant lignans are converted to enterolignans before uptake from the gut.

A method for sample preparation applied for both plasma [90] and urine [27] has been developed to facilitate lignan analysis using liquid chromatography with CEAD (LC–CEAD). Enzymatic hydrolysis with β‐glucuronidase is followed by LLE with a nonpolar solvent to extract the hydrolyzed conjugates. The extract is then loaded into a small‐scale ion‐exchange chromatography apparatus assembled in a Pasteur pipette packed with QAE‐Sephadex, from which the target analytes are then eluted with MeOH, while compounds more polar (ionized) than lignans are retained on the ion‐exchange material.

While performing research on the lignan composition of both mammalian and plant lignans of human urine, Nurmi et al. also tried some modifications of the enzymatic hydrolysis procedure [27]. The compounds in the *H. pomatia* extract cause a certain level of background noise during detection, and to reduce that, they tried to perform hydrolysis with pure β‐glucoronidase as well as with *H. pomatia* extract cleaned with active charcoal. Both methods gave worse yields compared to the impure extract, and subtracting the background noise from the extract was deemed necessary.

## 4 Lignan Analysis Using HPLC

### 4.1 Stationary Phases and Eluents

HPLC is the preferred methodology for the analysis of lignans from biological matrices. The main benefit of HPLC for the separation of medium‐polar molecules such as lignans is the relatively simple sample preparation needed. Even if the sample still contains a significant amount of matrix components, HPLC systems tend to be robust enough, as the column can simply be cleaned by running a strong organic solvent through it to make the column fit for analysis again. In HPLC, the compounds need no modification before analysis, while in GC analysis, lignans require a derivatization step before injection.

Another major advantage of HPLC is the various parameters that can be adjusted to modify the speed or resolution of the analysis. The column, mobile phase conditions, elution mode, and detector can all be changed to obtain the best possible results for the target analyte. Table 4 [13, 23, 27, 33, 61, 63, 70, 86, 88, 89, 90, 91, 92, 93] shows analytical HPLC methods used to quantify lignans from different samples. Due to the complexity of the samples in lignan analysis, gradient elution is necessary to accelerate the separation, as the samples will typically contain a wide range of molecules, from polar resin acids to highly lipophilic high molar mass compounds. Quantification is normally performed using external calibration curves, but internal standards can also be used when available. A variety of detectors have been used for quantification, with the best sensitivities provided by fluorescence detection (FLD) and even more sensitive MS methods.

**TABLE 4:** Liquid chromatographic quantifications of lignans in different samples.

Sample matrix	Column reversed‐phase	Gradient elution mobile phase	Quantification method	LOD	LOQ	Detection method	References
Biofluids	C18 (100 mm × 1 mm, 0.3 µm)	A: H_2_O + 0.1% HCOOH B: ACN + 0.1% HCOOH	Standard solutions IS glycine‐(1 × ^13^C)		0.0975 ng/mL	MRM QTRAP MS/MS	[89]
Coniferous knotwood	1: C18 (30 mm × 1.5 mm, 2.2 µm) 2: C18 (250 mm × 10 mm, 5 µm)	A: H_2_O + 0.1% HCOOH B: ACN + 0.1% HCOOH	Mixed standard solution	290 ng/mL	950 ng/mL	UV at 280 nm	[13]
Flax oil	C18 (150 mm × 4.6 mm, 1.8 µm)	A: H_2_O + 0.5% CH_3_COOH B: ACN	Standard solutions	50 ng/mL	180 ng/mL	800–50 scan TOF–MS	[86]
Foods	C18 (150 mm × 3 mm, 5 µm)	A: H_2_O B: MeOH	Standard solutions IS SECO‐d_8_, MATA‐d_6_	3.6 ng/mL		QQ–MS/MS	[70]
Human plasma	C18 (150 mm × 3 mm, 3 µm)	A: 50 mM NaOAc buffer pH 5/MeOH 80/20 B: NaOAc buffer/MeOH/ACN 40/40/20	Mixed standard solution		1.35 ng/mL	CEAD at 300, 420, 550 mV	[90]
Linden tree fruits	C18 (100 mm × 4.6 mm, 2.7 µm)	A: H_2_O + 0.01% HCOOH B: MeOH	Standard solutions	3.9 ng/mL		MRM MS/MS	[61]
Olive oil	C18 (250 mm × 4.6 mm, 5 µm)	A: H_2_O + 0.2% CH_3_COOH B: MeOH	Standard solutions		16 µg/kg	FLD excitation at 280 nm, emission at 313, 339, 353, 453 nm	[93]
Olive oil	Phenyl ether (250 mm × 4.6 mm, 4 µm)	A: H_2_O + 0.1% HCOOH B: MeOH/iPrOH 9/1 + 0.1% HCOOH	Standard solutions	0.116 µg/g	0.386 µg/g	UV at 280 nm for pinoresinol	[91]
Olive oil	C18 (150 mm × 4.6 mm, 1.8 µm)	A: H_2_O + 0.5% CH_3_COOH B: ACN	Standard solution + mix (IS DOPAC)	0.005 µg/mL	0.016 µg/mL	FLD; excitation at 285 nm, emission at 316, 328, 350, and 450 nm	[23]
Olives	C18 (250 mm × 4.6 mm, 5 µm)	A: H_2_O + HCOOH (pH 3.2) B: ACN	Standard solution standard: acetoxy‐pinoresinol		0.2 µg	ESI/MS	[92]
Persimmon leaves	C18 (250 mm × 4.6 mm, 5 µm)	A: ACN + 0.1% HCOOH B: H_2_O + 0.1% HCOOH	Mixed standard solution	25 ng/g		MRM and SRM QQQ–MS/MS	[33]
Urine	C18 (150 mm × 3 mm, 3 µm)	A: 50 mM NaOAc buffer pH 5/MeOH 80/20 B: NaOAc buffer/MeOH/ACN 40/40/20	Standard addition Mixed standard	0.67 ng/mL	3.37 ng/mL	300, 420, 620 mV CEAD	[27]
Wine	C18 (150 mm × 3 mm, 3 µm)	A: 50 mM NaOAc buffer pH 5/MeOH 80/20 B: NaOAc buffer/MeOH/ACN 40/40/20	Standard solutions	4.5 pg on column		420 mV CEAD	[88]
Wine, grapes	C18 (50 mm × 2.1 mm, 1.8 µm)	A: H_2_O + 0.01% CH_3_COOH B: MeOH	Standard solutions IS enterolactone		9 ng/mL	1700–100 scan Q–TOF–MS	[63]

Abbreviations: ACN, acetonitrile; CH_3_COOH, acetic acid; DOPAC, 3,4‐dihydroxyphenylacetic acid; HCOOH, formic acid; iPrOH, isopropanol; IS, internal standard; MATA‐d_6_, matairesinol‐d_6_; NaOAc, sodium acetate; SECO‐d_8_, secoisolariciresinol‐d_8_.

**FIGURE 4:**
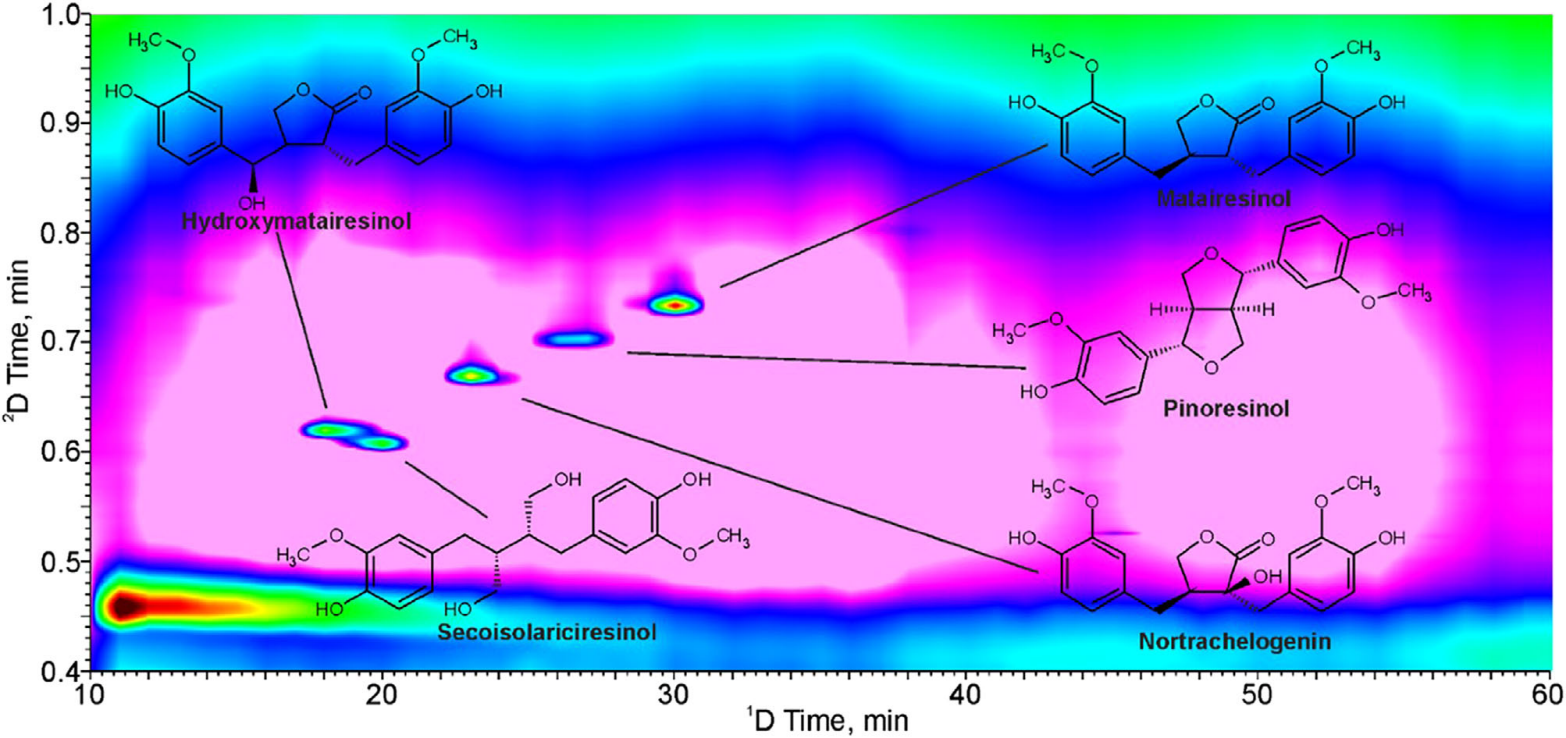
A 2D‐LC chromatogram from a lignan analysis of coniferous knotwood. Both columns used were C18 columns; in the first dimension, a Shim‐pack XR‐ODS II column was used, and in the second dimension, a Nucleodur C18 Pyramid column was used. The mobile phases used were A: water and B: ACN, both with 0.1% acetic acid, with D^1^ going from 15% to 60% B, and D^2^ from 20% to 90%, then back to 20% for re‐equilibration. Figure adapted from Falev et al. [13], licensed under Creative Commons Attribution License (https://creativecommons.org/licenses/by/4.0/).

The choice of the stationary phase is most important for optimization of the analysis. The most common choice of columns for lignan analysis is reversed‐phase silica columns (C8 or C18). Organic molecules in the sample have affinity toward these lipophilic chains and are retained based on their polarity; the less polar the molecule is, the more strongly it is retained. As biomolecules are often produced through enzymatic processes in nature, certain enantiomers of chiral compounds sometimes appear to be favored over others. To investigate the enantiomeric excess in plant samples, chiral LC columns based on proteins, sugars, cyclodextrins, polysaccharides, or other large organic molecules providing multiple chiral centers have been used [68, 94, 95]. Two‐dimensional LC × LC has also been utilized in lignan analyses. For example, Falev et al. employed LC × LC, with identical columns, to quantify lignans from coniferous knotwood that would have overlapping peaks in one‐dimensional LC–UV. A typical chromatogram is shown in Figure 4 [13].

Isocratic elution is often used for prep‐LC to keep the elution time of the collected peaks as stable as possible and to avoid contamination of the desired products [82, 83]. While isocratic elution is not typically used for the analysis of non‐chiral samples, it is employed for chiral determinations. As enantiomers are difficult to separate and require long retention times to achieve baseline separation, the percentage of organic solvent is kept low and constant [68]. As typical lignan samples contain compounds of varying polarities, separation of these compounds is mostly performed using gradient elution [1]. Starting from a mostly aqueous mobile phase, the percentage of organic solvent is gradually increased until the mobile phase is mostly or completely organic. By doing so, an optimized method can be created for the specific separation at hand, which will save analysis time while possibly maintaining the baseline separation of the compounds.

### 4.2 Detectors

**FIGURE 5:**
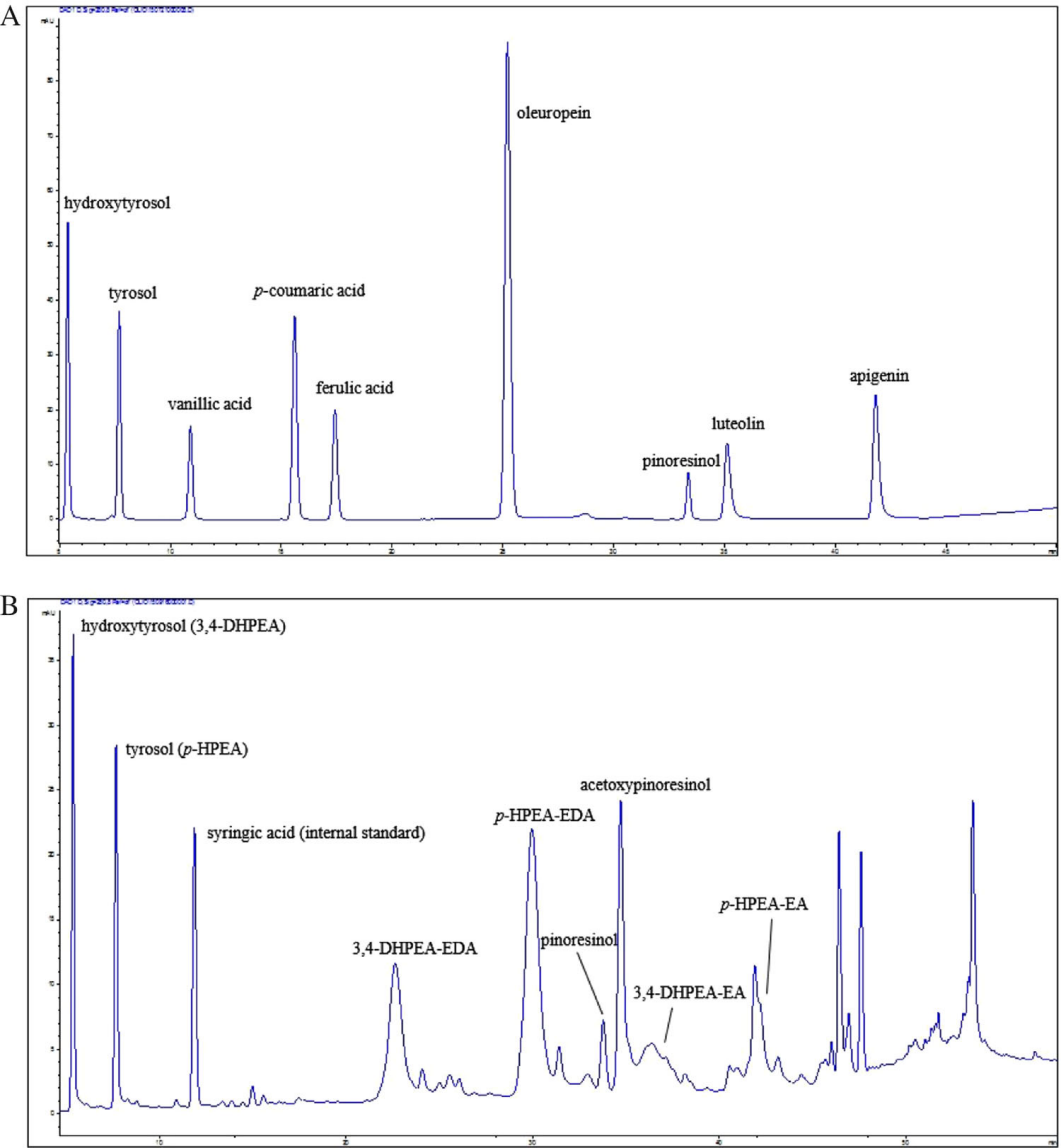
Analysis of polyphenols in olive oil by HPLC–UV/Vis analysis. Separation of compounds in (A) a standard mixture and (B) in an olive oil sample. The separation was performed on a Phenomenex Synergi Polar‐RP column, with mobile phases consisting of (A) water + 0.1% formic acid and (B) 90/11 MeOH/*i*PrOH + 0.1% formic acid. Figure adapted from Ricciutelli et al. [91] with permission from Elsevier.

The most common detector for lignan analysis is a UV/Vis spectrometer, which measures the absorbance of molecules in the ultraviolet and visible regions. The most commonly used wavelength for lignans is 280 nm, but by using diode array detectors (DADs), more wavelengths can be monitored at any given time, as 210 and 230 nm can also give a decent response. This detection method is more commonly used for qualitative separations than quantification due to the lack of sensitivity of UV/Vis. Multiple different matrices have been used to identify lignans by HPLC–UV/Vis, and the method has been used as a complementary method to LC‒MS for the characterization of wood samples, such as the bark of Scots pine [10] and the branches of Norway spruce [72]. Characterization of food samples such as olives [92, 96], olive oil [84, 87], pomegranate [65], and flaxseed [74] has been performed using LC–UV. Quantification of lignans by UV‒Vis has also been used. Pinoresinol and other lignans were quantified from softwood samples using a DAD/UV detector connected to an LC × LC system, which was also linked to a fraction collector, so the detected lignans could be collected for NMR characterization [13]. HPLC–DAD/UV was also used to quantify polyphenols from samples of extra‐virgin olive oil [91]. They used several different wavelengths for quantifying different compounds, with 280 nm being the wavelength selected for pinoresinol and four other compounds, as shown in Figure 5.

Since lignans are phenolic compounds containing two aromatic rings, they can be detected by FLD. As only certain kinds of molecules can be detected using FLD, the technique is more selective and sensitive than UV/Vis, as matrix components play a much smaller role. FLD has mainly been used to quantify lignans in, for example, olive oil and flaxseed [97]. Selvaggini et al. studied the contents of phenolic compounds of three different olive cultivars while comparing the performance between UV and FLD quantifications [93]. They found that, for all compounds except for one, FLD provided lower quantitation limits and thus better performance. While also measuring phenolic compounds in olive oils, Monasterio et al. compared FLD and MS detection for their target compounds [23]. In general, ion trap MS provided lower limits of quantitation, but for pinoresinol, FLD proved to be more sensitive than MS, albeit within an extremely narrow linear range.

**FIGURE 6:**
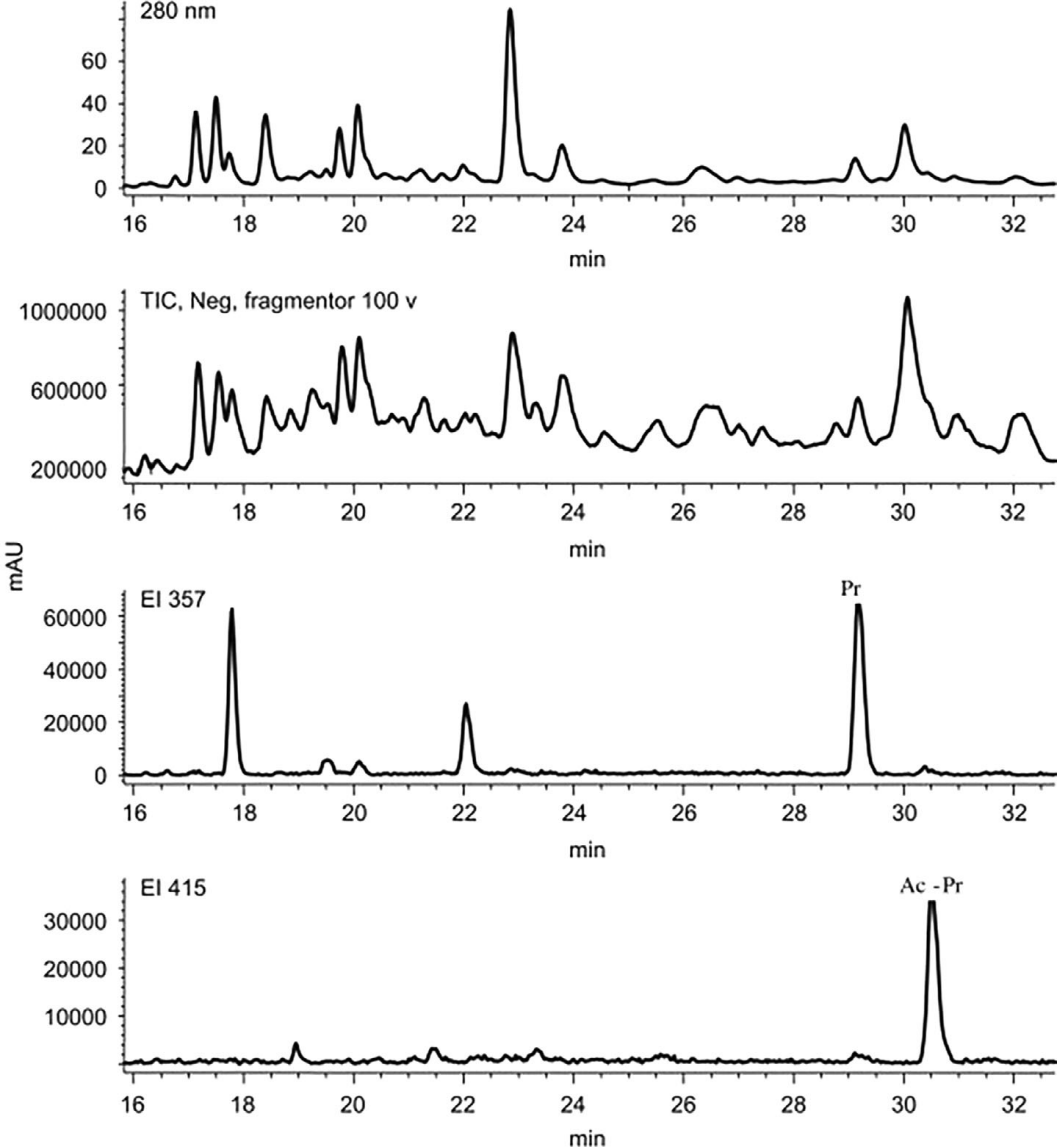
HPLC–UV/MS chromatogram of the ether fraction from the methanol extract at reflux from powdered olive stones from Taggiasca, Italy. A Luna RP18 column was used, with mobile phases A: water + 0.1% formic acid and B: ACN. The total‐ion chromatogram (TIC) shows all the compounds in the sample, while the extracted ion current (EIC) graphs show when peaks with those specific *m*/*z* values (EI 357 and EI 415) elute. Figure adapted from Oliveras López et al. [92] with permission from Elsevier.

MS is the most common detector for the quantification of lignans. A useful feature of the MS detector is demonstrated in Figure 6, where the total‐ion chromatogram (TIC) shows all the compounds in the sample, but it is hard to identify which individual compounds are present [92]. Because MS records individual spectra for each peak shown in the TIC, it is possible to filter out any mass‐to‐charge ratios that do not correspond to target analytes, giving extracted ion current (EIC) chromatograms, which enable compound identification in the sample. LC‒MS has been used to identify and quantify lignan compounds from various matrices, including food samples, such as fruits [19, 61, 63, 92] and food oils [86, 93], plants [98, 99], as well as quantification from biofluid samples. MS has also been used to characterize unknown lignans from samples, as mass‐to‐charge ratios and fragmentation patterns are identifiers that can be used to determine unknown molecules [64]. Even in studies where HPLC separations have not been used, MS detection has been employed in direct injection mode [77, 100].

**FIGURE 7:**
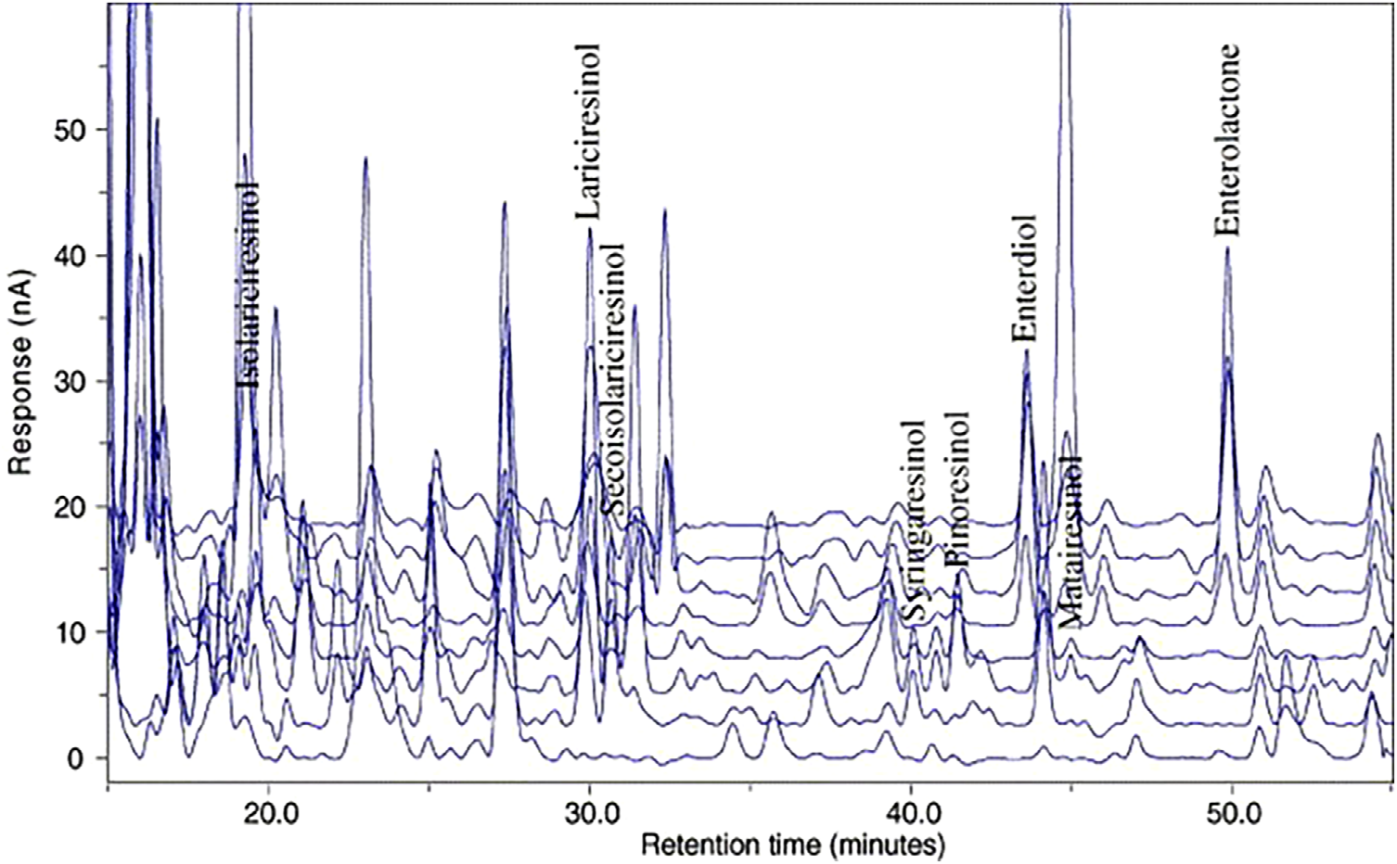
HPLC–CEAD chromatogram from a urine sample lignan analysis. The column used for the separation was an Inertsil ODS‐3 reversed‐phase column equipped with an Inertsil ODS‐2 precolumn. A gradient method with mobile phases of A: 50 mM sodium acetate buffer at pH 5/MeOH 80/20 (v/v) and B: buffer/MeOH/ACN 40/40/20 (v/v/v) was used to separate the lignans. Figure adapted from Schroeder et al. [25] with permission from Elsevier.

CEAD is a less common but sensitive method for detecting analytes in HPLC. Typical LODs are in the range of 0.5–2.5 µg/L for complex matrices (in the low pg to fmol range on‐column), as also seen in Table 4. The voltage used is highly dependent on the compound studied, and for example, for pinoresinol, the voltage giving a maximum signal used for quantification is 420 mV. LC–CEAD has been used to study the content of lignans from various liquid samples, such as wine [88], urine [27], and plasma [90], and a typical analysis of a urine sample is shown in Figure 7. Of note is the detection of plant lignans found in biological samples, as they had not all been converted to the mammalian lignans enterodiol and enterolactone [27]. These studies generally show the excellent sensitivity of the method, as the obtained limits of detection and quantitation are on par with sensitive MS‐based detectors. Solid samples have also been studied using CEAD after simple extractions, such as extracts from legume and sweet chestnut flours [32].

## 5 Conclusions

This review provides an overview of the methods for isolation and analysis of lignans. The extraction of lignans is mostly performed using conventional techniques with aqueous alcohol solutions. While more advanced methods, accelerating the extraction, such as microwaves, sonication, or pressure, are useful for cutting down the time required for the extraction, most lignan samples will require lengthy purification procedures afterward, regardless. Especially for the isolation of compounds, conventional extractions are emphasized with the advantage of being able to process larger amounts of sample in one go. The purification procedures for lignans typically start with a hydrolysis step to coax the target molecules out of the larger lignin structures commonly found in the samples. For less complex samples, a simple LLE procedure is often enough to analyze the lignan content in samples, but when quantifying compounds in complex biological matrices, RP‐SPE and silica gel flash chromatography are necessary to isolate the target molecules. The most common analytical technique used in lignan analysis is LC. Despite the very complex structures of the samples, memory effects have typically not been an issue. While it is possible to use GC for this purpose, lignan samples require derivatization steps for the molecules to be separated by GC. A wide variety of detectors are viable in lignan analysis, with UV being fast and easy, MS providing excellent performance and utility, and the less commonly used CEAD detectors also showing remarkably low limits of quantification.

For future prospects in lignan analysis, an increase in the availability and selection of deuterated lignan internal standard materials could provide increased accuracy in the results of quantitative lignan analysis. Currently, only a few deuterated lignan standards are available, and for those that are, the prices are often well outside the ranges of academic budgets. The increasing interest in natural products, such as lignins, with a large number of health benefits, will push this area forward.

## Author Contributions

**Miikka Paloluoto**: conceptualization, data curation, formal analysis, investigation, methodology, visualization, writing – original draft. **Susanne K. Wiedmer**: conceptualization, funding acquisition, project administration, supervision, writing – original draft, writing – review and editing

## Conflicts of Interest

The authors declare no conflict of interest.

## Data Availability

The authors confirm that the data supporting the findings of this study are available within the article.
